# Supplementary material for: Effects of calibrated blue–yellow changes in light on the human circadian clock
Source: Nat Hum Behav. 2023 Dec 22;8(3):590–605. doi: 10.1038/s41562-023-01791-7 (PMC10963261; doi:10.1038/s41562-023-01791-7)
Supplement: Supplementary file 1 — Supplementary Figs. 1–6, methods, results, Tables 1–23, Picture 1, laboratory log, example protocol and references. [file 41562_2023_1791_MOESM1_ESM.pdf]

nature human behaviour

Registered Report https://doi.org/10.1038/s41562-023-01791-7

Effects of calibrated blue–yellow changes in light on the human circadian clock

In the format provided by the authors and unedited

Supplementary Material S1.

Table of Contents

	Page
Supplementary Figures	2
Supplementary Methods	8
Supplementary Results	9
Supplementary Tables	10
Supplementary Picture	24
Laboratory Log	25
Example Protocol	26
Supplementary References	27

Supplementary Figures

Secondary Outcomes.

Melatonin Concentrations (S1).

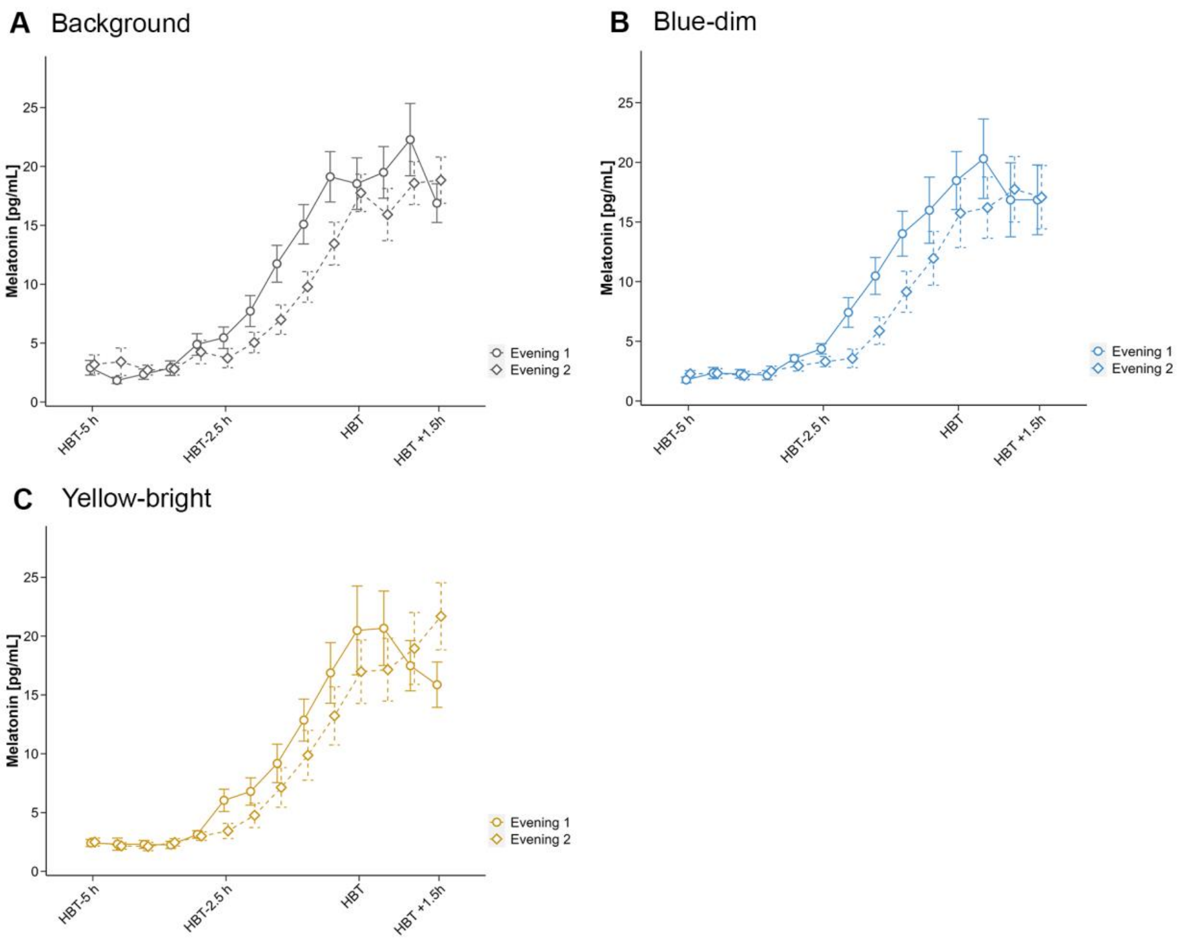

Supplementary Figure 1. Melatonin concentrations. Time course of melatonin concentrations during the first (solid line) and second (dashed line) evening in the laboratory in each condition (A “background”, B “blue-dim”, C “yellow-bright”). We show the mean with error bars representing the standard error. Analyses were based on data from 16 participants who underwent 3 experimental conditions.

Visual Comfort (S4). Visual comfort was calculated as the average rating from the responses to the questions about how pleasant the lighting was generally, how participants perceived the level of brightness, how glaring the light source was, and how pleasant participants rated the colour temperature. The inspection of the data showed that the assumptions for mixed linear models were met (cf. Supplementary Methods for more details). There was moderate (inconclusive) evidence against a difference between the conditions regarding visual comfort experienced during the light exposure (i.e., how pleasant/ bright/ glaring the light was, and how warm/cold the light colour was). The data was approximately 6 times more likely under the H0

than under the H1 (BF_{10} = 0.16). Suppl. Table **12** provides an overview of the condition mean (intercept) and deviations from the intercept sampled from the posterior distribution. For the condition × time interaction, there was moderate evidence in favour of H1 (BF_{10} = 7.68). Supplementary Figure 2 shows the time course of visual comfort ratings.

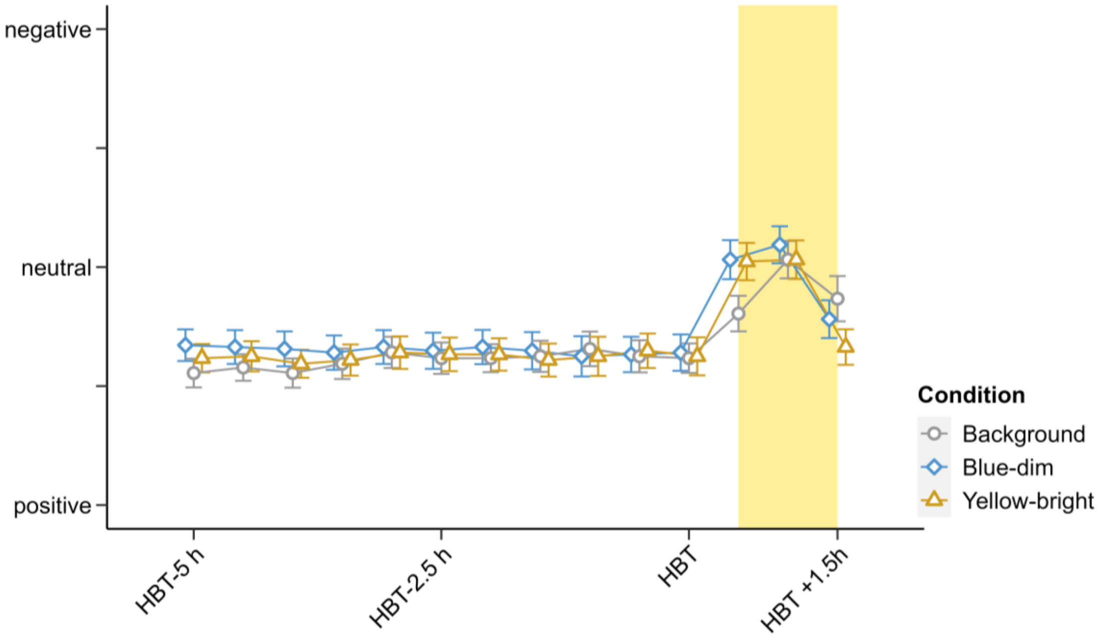

Supplementary Figure 2: Visual comfort ratings. Mean visual comfort scores during the first evening in the laboratory. Error bars indicate the standard error. The yellow box indicates the period of the light exposure. HBT = Habitual bedtime. Analyses were based on data from 16 participants who underwent 3 experimental conditions.

PVT: Median Reaction Time (RT; S5), Fastest 10% RTs (S6), Slowest 10% RTs (S7).

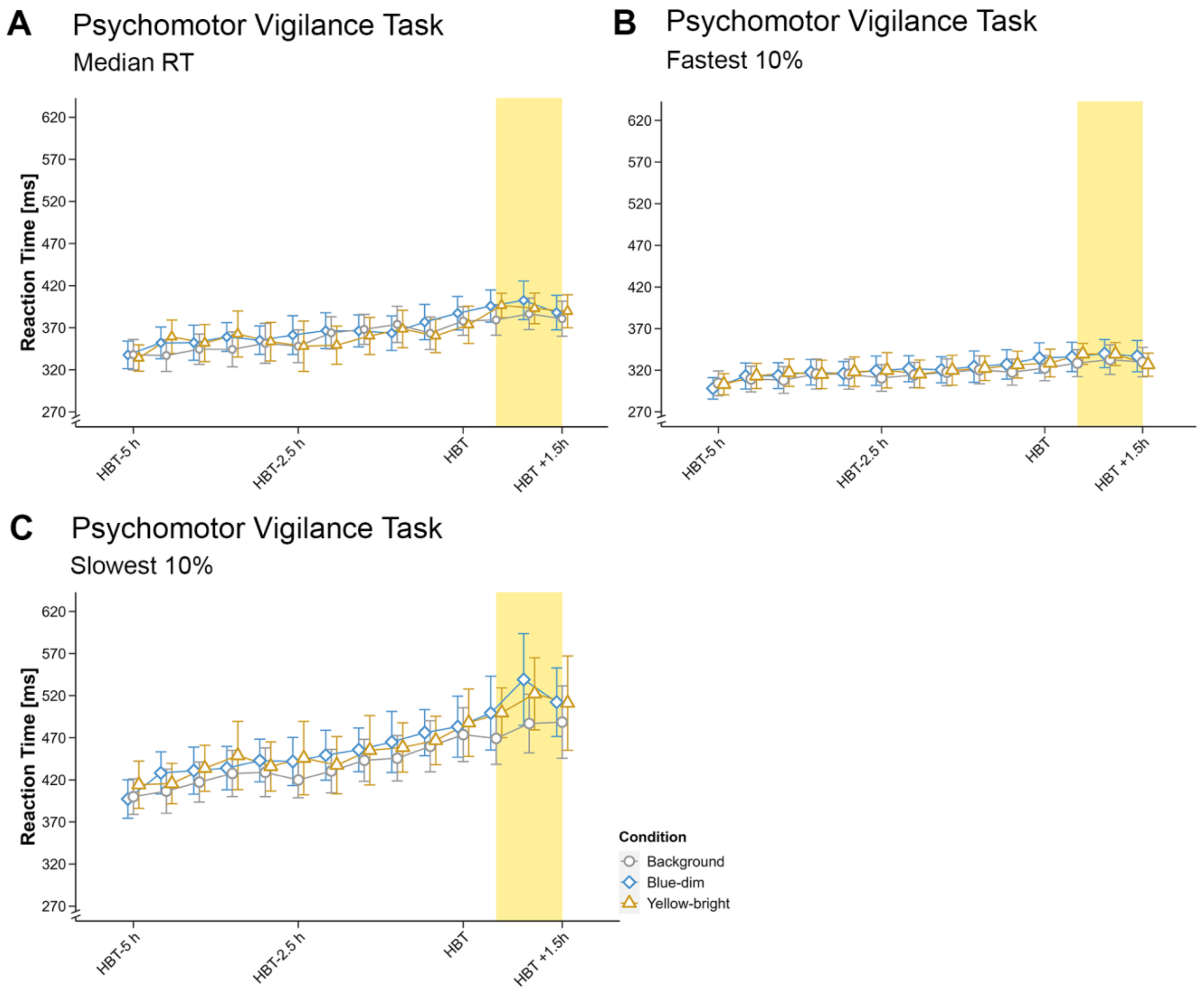

Supplementary Figure 3: Reaction times on the psychomotor vigilance task (PVT). A Median, B mean of the 10% fastest, C mean of the 10% slowest reaction times across the first evening in the laboratory. Error bars indicate 95% confidence intervals. The yellow box indicates the duration of the light exposure. Analyses were based on data from 16 participants who underwent 3 experimental conditions. HBT = Habitual bedtime.

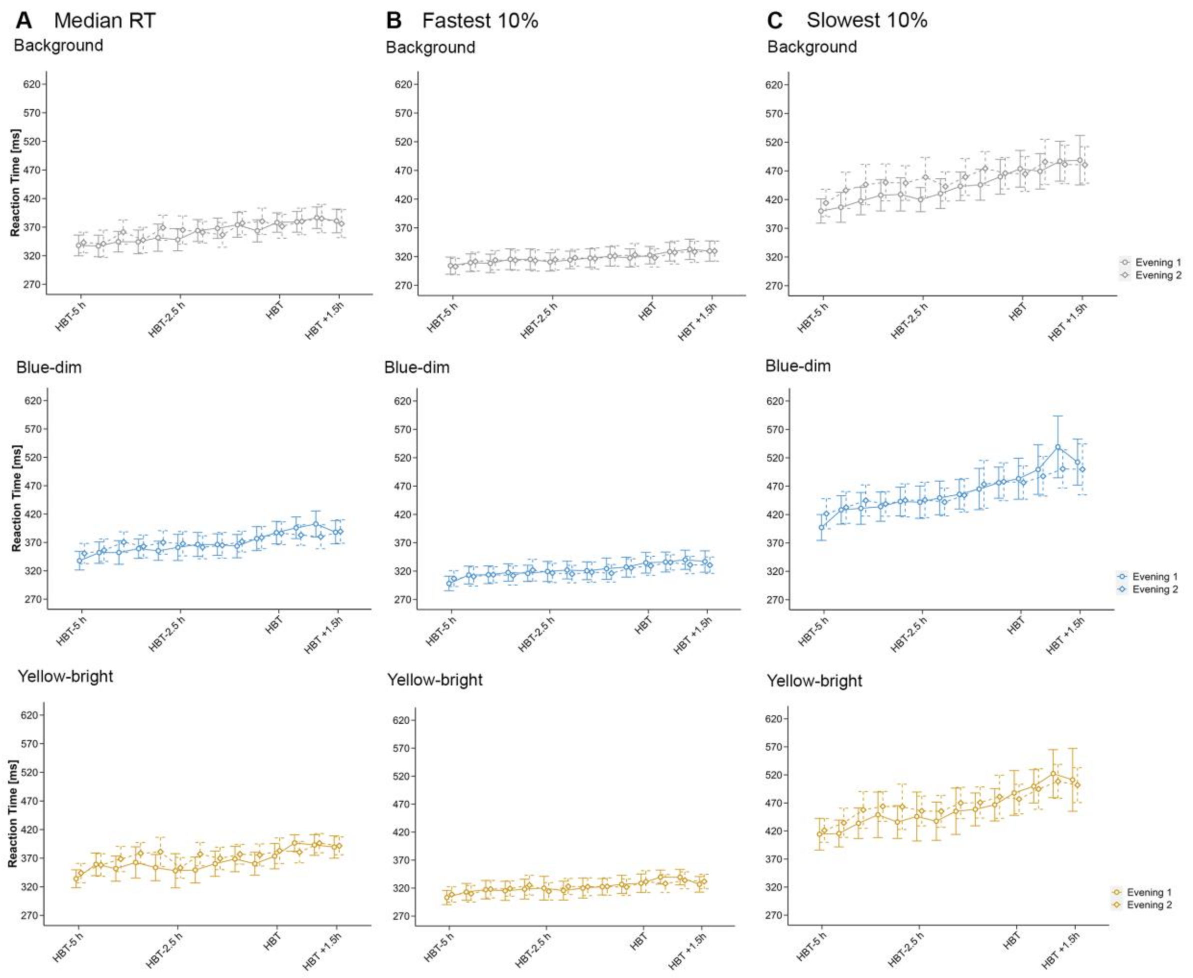

Supplementary Figure 4: Reaction times on the psychomotor vigilance task (PVT). A Median, B mean of the 10% fastest, C mean of the 10% slowest reaction times across the first (solid lines, circles) and second (dashed lines, diamonds) evenings in the laboratory. Error bars indicate 95% confidence intervals. Note that the data from the second evening were not part of the analysis plan and are only shown here for completeness. We thus also refrain from statistical analyses. Results shown are based on data from 16 participants who underwent 3 experimental conditions. HBT = Habitual bedtime.

EEG-derived Sleep Onset Latency (SLAT; S8).

The inspection of the data indicated that the assumptions for mixed linear models were met, and rank-transformation did not increase compatibility with the assumptions (cf. Supplementary Methods for more details). Analyses yielded inconclusive evidence against a condition difference regarding the onset latency to 10 minutes of continuous sleep. The data were approx. 3 times more likely given the H0 than the H1 (BF_{10} = 0.31). The mean latency to 10 minutes of continuous sleep was 17.0 ± 42.2 minutes in the background, 9.4 ± 13.2 minutes in the yellow-bright, and 7.5 ± 4.3 minutes in the blue-dim condition. An overview of the condition means (intercept) and standard deviations sampled from the posterior distribution is provided in Suppl. Table **16**. Suppl. Fig. **5** below provides an illustration of the results. For a

comprehensive overview of the sleep data for each light condition and visit, see Suppl. Table **17**.

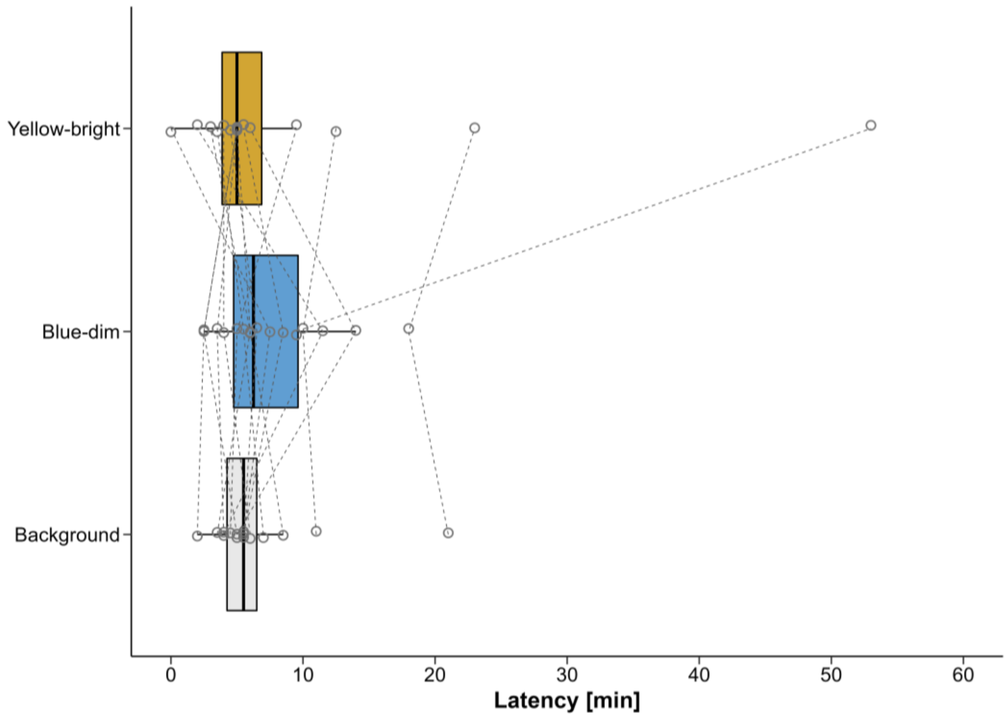

Supplementary Figure 5: Latency to 10 minutes of continuous sleep. In the boxplots, the lower and upper hinges of the boxplot correspond to the 25% and 75% quartiles, the thick black line indicates the median. Whiskers extend to the lowest/largest value at most 1.5× the interquartile range (IQR) from the hinges. Gray circles represent individual values of participants. Note: The individual data point from one participant, who had a latency of 174.5 min to 10 min of continuous sleep in the background condition, was removed from the plot as this would have concealed the pattern of the other data points. Analyses were based on data from 16 participants who underwent 3 experimental conditions.

Subjective Sleepiness (S2).

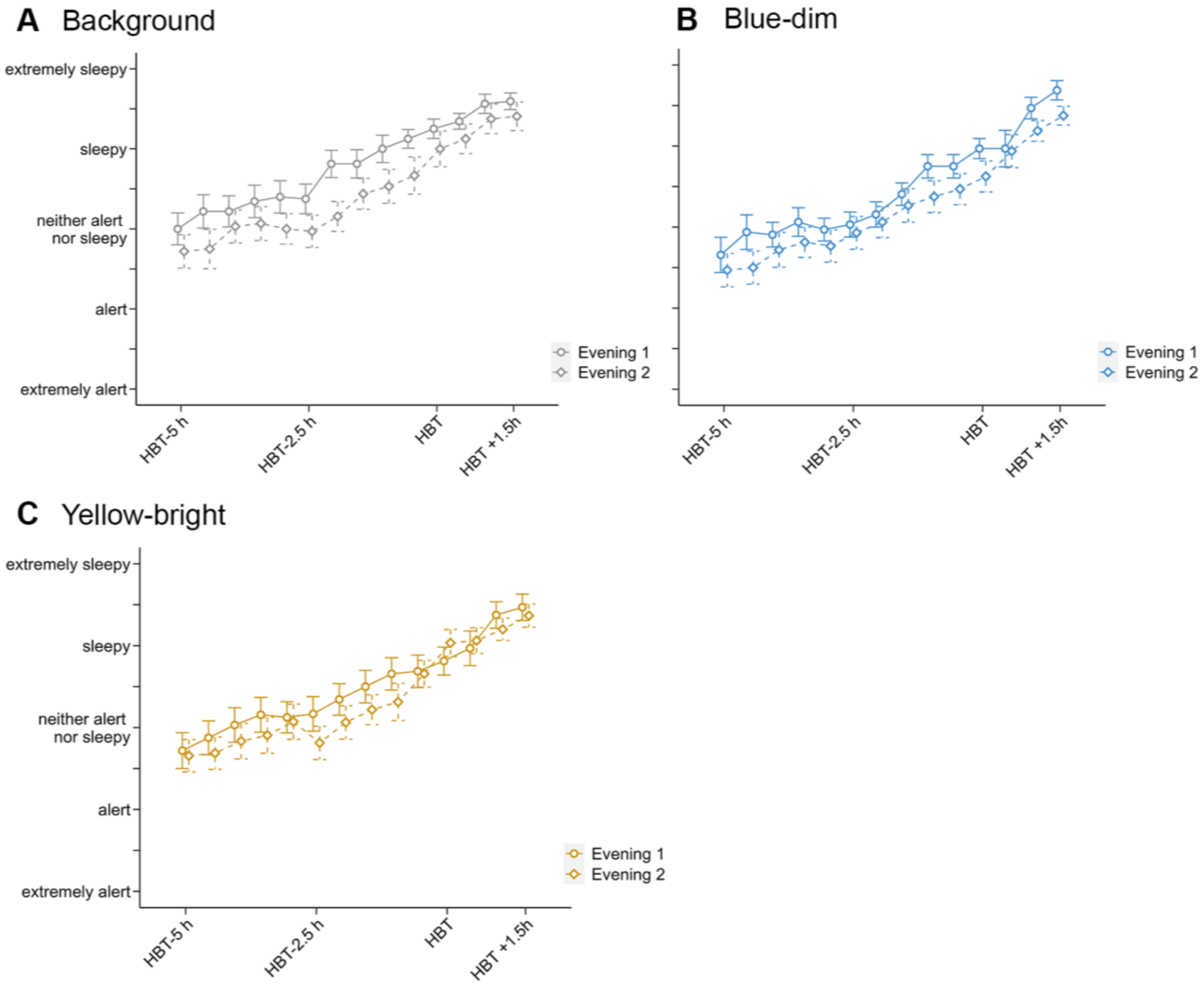

Supplementary Figure 6: Subjective Sleepiness. Mean and standard error of subjective sleepiness as assessed with the Karolinska Sleepiness Scale (KSS) during evening 1 (solid lines, circles) and evening 2 (dashed lines, diamonds) in the three light exposure conditions. Note that the data from the second evening were not part of the analysis plan and are only shown here for completeness. We thus refrain from statistical analyses. Displayed results are based on data from 16 participants who underwent 3 experimental conditions. HBT = Habitual bedtime.

Supplementary Methods

Protocol. The reason to not fully randomise the order of conditions was that a full randomisation would have required us to acquire data from multiples of 12 participants (i.e., always 6 men and 6 women). Given our financial and resource limit of 16 participants, this would not have worked out.

Statistical data analysis. For all analyses, we report BF_{10}, i.e., the likelihood of the data under H1 compared to H0. We inspected whether the assumptions of linear mixed models were met as follows: (i) correctness of the model equation, i.e., linearity, by plotting residuals against the explanatory variable, and (ii) constant variance of errors (homoscedasticity) was assessed by plotting residuals against fitted values. Further, we assessed the (iii) normality of the residuals using QQ-plots and density plots of the residuals. We also explored, whether rank-transformation of the data would improve the compatibility with the model assumptions. If this was the case, we report results for both analyses. Following general recommendations[1], error percentages below 20% were deemed acceptable (default: 10 000 iterations) as this should result in the same qualitative conclusion.

Data acquisition. Data acquisition took place between March and December 2022 with a break in August. More precisely, the following number of volunteers (in brackets) had their first experimental visit in each month: March (1), April (2), May (0), June (2), July (2), September (1), October (3), November (3), December (2). The Laboratory Log provides even more detailed information on when each participant had his/her visits, cf. https://doi.org/10.6084/m9.figshare.23578695 or below.

Supplementary Results

Subjectively reported light history. The following table gives an overview (mean ± SD; range) of the reported light history on the day the volunteers came to the lab.

	Time under open sky [in hours]	Light quality	Amount of light
**Visit 1**	1.6 ± 1.2 (0.5-4.5)	6.4 ± 2.7 (1-10)	6.9 ± 2.3 (2-10)
**Visit 2**	1.5 ± 1.3 (0.3-5.3)	6.8 ± 2.3 (1-10)	5.2 ± 1.8 (2-8)
**Visit 3**	1.7 ± 1.5 (0.5-5.3)	6.1 ± 2.4 (3-10)	5.8 ± 2.1 (3-10)

Comments. Light quality and the amount of light that reached the eyes was assessed using Likert scales (range 0-10; 0 = very dull day/ very little light, 10 = bright summer day/ a lot of light).

Supplementary Tables

Suppl. Table 1. Converging evidence for a role of S-cone-opponent circuitry.

Study	Species	Level of Analysis	Findings
Mouland et al. (2019)[2]	Rodents	Behavioural	• Light modulations, which stimulate the short wavelength-sensitive S and long wavelength-sensitive L cones in an opponent fashion affect the murine circadian system differentially: Yellow-bright stimuli have stronger circadian phase-shifting effects than blue-dim stimuli.<br>• Shows that these modulations have effects independent of the melanopsin system.
Dacey et al. (2005)[3]	Primates	Anatomical	• S-cones provide an inhibitory input and the L and medium wavelength-sensitive M cones provide an excitatory input to the ipRGCs.
Patterson et al. (2020)[4]	Primates	Anatomical	• The circuit described by Dacey and colleagues is due to a specific amacrine cell.
Krauskopf et al. (1982)[5]<br>Webster et al. (1994)[6]	Humans	Psychophysical	• Colour vision is organised by so-called ‘cardinal directions’, one of which is the S–[L+M] direction pitting S-cones against luminance (L+M)
Spitschan et al. (2014)[7]<br>Cao et al. (2015)[8]<br>Woelders et al. (2018)[9]	Humans	Physiological	• The pupil light response, which is fed by the ipRGCs receives an S-cone opponent input.<br>• This also confirms the circuit described by Dacey and colleagues.
Figueiro et al. (2004)[10]	Human	Neuroendocrine	• Both light sources, one with a peak at 460 nm (blue LED) and one without (mercury vapor, yellowish light), elicit melatonin suppression. The blue LED is more effective.<br>• Supports the role of S-cone-opponent signals in melatonin suppression in humans.

Suppl. Table 2. Design Table.

Question	Hypothesis	Sampling plan	Analysis Plan	Interpretation given to different outcomes
**Primary Outcome**				
**Dim-light melatonin onset (DLMO) (*C1*)**	The difference in DLMO (in minutes) between evenings 1 and 2 of the protocol are larger in the yellow-bright condition than in the blue-dim condition. The DLMO difference in both conditions is larger than in the constant light condition.<br>Effect of interest: condition	Sequential Bayes Factors (N_{min} = 4; N_{max} = 16)	Repeated-measures ANOVA<br>Within-subject factor: condition<br>Random factors: participant ID, gender	<u>Inconclusive evidence</u><br>1<BF<3 = anecdotal evidence for H0/H1<br>1<BF<10 = moderate evidence for H0/H1<br><u>Conclusive evidence</u><br>10<BF<30 = strong evidence for H0/H1<br>BF>30 = very strong evidence for H0/H1
**Secondary Outcomes**				
**Melatonin concentrations (*S1*)**	The time course (t1, t2, …tn) of melatonin concentrations on evening 1 of the protocol will differ between the three conditions. Concentrations in the constant light condition will be highest, in the yellow-bright flickering they will be lowest, the blue-dim flickering will be associated with intermediate melatonin levels.<br>Effects of interest: condition, time × condition interaction.	See sampling plan of C1	Repeated-measures ANOVA<br>Within-subject factors: condition, time<br>Random factors: participant ID, gender	See interpretation of C1
**Subjective sleepiness (*S2*)**	The time course (t1, t2, …tn) of subjective sleepiness ratings on the Karolinska Sleepiness Scale (KSS) will differ between the three conditions. KSS values in the constant light condition will be highest (indicating higher sleepiness), in the yellow-bright flickering they will be lowest, the blue-dim flickering will be associated with intermediate KSS values.<br>Effect of interest: condition, time × condition interaction.	See sampling plan of C1	Repeated-measures ANOVA<br>Within-subject factors: condition, time<br>Random factors: participant ID, gender	See interpretation of C1

**Objective Sleepiness (*S3*)**	The time course (t1, t2, t3) of EEG-derived alpha (8-12 Hz)/ theta (4-7 Hz) ratio values will differ between the three conditions. Ratios in the constant light condition will be smallest (indicating higher sleepiness), in the yellow-bright flickering they will be highest, the blue-dim flickering will be associated with intermediate ratios.<br>Effects of interest: condition, time × condition interaction.	See sampling plan of C1	Repeated-measures ANOVA<br>Within-subject factors: condition, time<br>Random factors: participant ID, gender	See interpretation of C1
**Visual comfort (*S4*)**	The time course (t1, t2, …tn) of visual comfort ratings will differ between the three conditions. Visual comfort ratings in the constant light condition will be highest (indicating higher comfort), in the yellow-bright flickering they will be lowest, the blue-dim flickering will be associated with intermediate visual comfort values.<br>Effect of interest: condition, time × condition interaction.	See sampling plan of C1	Repeated-measures ANOVA<br>Within-subject factors: condition, time<br>Random factors: participant ID, gender	See interpretation of C1
**PVT: Median reaction time (RT) (*S5*)**	The time course (t1, t2, …tn) of median RTs will differ between the three conditions. Median RTs in the constant light condition will be highest, in the yellow-bright flickering they will be lowest, the blue-dim flickering will be associated with intermediate median RTs.<br>Effect of interest: condition, time × condition interaction.	See sampling plan of C1	Repeated-measures ANOVA<br>Within-subject factors: condition, time<br>Random factors: participant ID, gender	See interpretation of C1
**PVT: Fastest 10% RTs (*S6*)**	The time course (t1, t2, …tn) of the 10% fastest RTs will differ between the three conditions. 10% fastest RTs in the constant light condition will be highest, in the yellow-bright flickering they will be lowest, the blue-dim flickering will be associated with intermediate 10% fastest RTs.<br>Effect of interest: condition, time × condition interaction.	See sampling plan of C1	Repeated-measures ANOVA<br>Within-subject factors: condition, time<br>Random factors: participant ID, gender	See interpretation of C1
**PVT: Slowest 10% RTs (*S7*)**	The time course (t1, t2, …tn) of the 10% slowest RTs will differ between the three conditions. 10% slowest RTs in the constant light condition will be highest, in the yellow-bright flickering they will be lowest, the blue-dim flickering will be associated with intermediate 10% slowest RTs.	See sampling plan of C1	Repeated-measures ANOVA<br>Within-subject factors: condition, time	See interpretation of C1

	Effect of interest: condition, time × condition interaction.		Random factors: participant ID, gender	
**EEG-derived sleep onset latency (SLAT)** ***(S8)***	The SLAT (in minutes) on evening 1 of the protocol is longer in the yellow-bright condition than in the blue-dim condition. The SLAT in both conditions are longer than in the constant light condition.<br>Effect of interest: condition	See sampling plan of C1	Repeated-measures ANOVA<br>Within-subject factor: condition<br>Random factors: participant ID, gender	See interpretation of C1
**EEG-derived slow wave activity (SWA) (*S9*)**	The time course (t1, t2, …tn) of SWA (averaged power between 0.5 and 4.5 Hz at electrodes F3, F4, Fz) in each percentile of the first sleep cycle will differ between the three conditions. SWA in the constant light condition will be highest, in the yellow-bright flickering they will be lowest, the blue-dim flickering will be associated with intermediate median RTs.<br>Effect of interest: condition, time × condition interaction.	See sampling plan of C1	Repeated-measures ANOVA<br>Within-subject factors: condition, time<br>Random factors: participant ID, gender	See interpretation of C1
**Outcome-neutral measurements**				
**Melatonin concentrations (*ON1*)**	The time course (t1, t2, …tn) of melatonin concentrations on evening 1 of the protocol before the light exposure will *not* differ between the three conditions.<br>Effects of interest: condition, time × condition interaction.	See sampling plan of C1	Repeated-measures ANOVA<br>Within-subject factors: condition, time<br>Random factors: participant ID, gender	See interpretation of C1
**PVT: Median reaction time (RT) (*ON2*)**	The time course (t1, t2, …tn) of median RTs before the light exposure will *not* differ between the three conditions.<br>Effects of interest: condition, time × condition interaction.	See sampling plan of C1	Repeated-measures ANOVA<br>Within-subject factors: condition, time<br>Random factors: participant ID, gender	See interpretation of C1

**Subjective sleepiness (*ON3*)**	The time course (t1, t2, …tn) of subjective sleepiness ratings on the Karolinska Sleepiness Scale (KSS) before the light exposure will *not* differ between the three conditions.<br>Effect of interest: condition, time × condition interaction.	See sampling plan of C1	Repeated-measures ANOVA<br>Within-subject factors: condition, time<br>Random factors: participant ID, gender	See interpretation of C1
**Brightness (*ON4*)**	The time course (t1, t2, …tn) of perceived brightness ratings before the light exposure will *not* differ between the three conditions.<br>Effect of interest: condition, time × condition interaction.	See sampling plan of C1	Repeated-measures ANOVA<br>Within-subject factors: condition, time<br>Random factors: participant ID, gender	See interpretation of C1

Factors: “condition” = three factor levels (constant light, blue-dim flickering, yellow-bright flickering); “time” = multiple assessments/time course within one condition (factor levels: t1, t2, …, tn); “gender” = two factor levels (man vs. woman); “participantID” = participant code (number of factor levels depends on final sample size); BF = Bayes Factor, can be BF_{10} or BF_{01}. Note that we decided to implement the repeated-measures ANOVA using a linear model approach to circumvent the issue of case-wise deletion in case of missing data.

Suppl. Table 3. Overview of the derived measure and the number of outcome measurements per participant for each hypothesis in the design table (Table 1).

Measurement modality	Derived measure	Number of outcome measurements per participant
**Primary outcome**		
**Melatonin concentrations (*C1*)**	*Circadian phase shifts*	3 (1x constant light, 1x 'blue-dim' flickering, 1x 'yellow-bright' flickering)
Secondary outcomes		
**Melatonin concentrations (*S1*)**	*Time series of melatonin concentrations*	42 (14x constant light, 14x 'blue-dim' flickering, 14x 'yellow-bright' flickering)
**Subjective sleepiness (*S2*)**	*Time series of subjective sleepiness ratings*	42 (14x constant light, 14x 'blue-dim' flickering, 14x 'yellow-bright' flickering)
**Objective Sleepiness (*S3*)**	*Time series of objective sleepiness*	9 (3x constant light, 3x 'blue-dim' flickering, 3x 'yellow-bright' flickering)
**Visual comfort (*S4*)**	*Time series of visual comfort ratings*	30 (10x constant light, 10x 'blue-dim' flickering, 10x 'yellow-bright' flickering)
**Reaction time (*S5*)**	*Time series of median RT*	42 (14x constant light, 14x 'blue-dim' flickering, 14x 'yellow-bright' flickering)
**(*S6*)**	*Time series of fastest 10% RT*	42 (14x constant light, 14x 'blue-dim' flickering, 14x 'yellow-bright' flickering)
**(*S7*)**	*Time series of slowest 10% RT*	42 (14x constant light, 14x 'blue-dim' flickering, 14x 'yellow-bright' flickering)
**Sleep EEG (*S8*)**	*Sleep onset latency to continuous 10 min of sleep (SLAT)*	3 (1x constant light, 1x 'blue-dim' flickering, 1x 'yellow-bright' flickering)
**(*S9*)**	*Slow Wave Activity (SWA)*	30 (10 percentiles 'constant light', 10 percentiles 'blue-dim', 10 percentiles 'yellow-bright')

Suppl. Table 4. Overview of the irradiance-derived α-opic responses (Ee; in mW/m2) as well as irradiance-derived chromaticity values (CIE 1931 xy standard observer for a 2° field) for the ambient light at different locations in the room, that is, the situation during the day and between assessments in the evening. Measurements were taken from the observer's point of view. Values were calculated using the luox app[11,12]. For a picture of the setup, please see Suppl. Photo 1.

	α-opic irradiances [mW/m^2]				CIE 1931 xyY		
	L cones	M cone	S-cone	Melanopsin	x	y	Illuminance [lx]
At desk with display (1)	13.04	8.45	1.05	3.54	0.49	0.42	7.82
On the sofa facing away from the ambient light (2)	10.05	6.54	0.79	2.74	0.49	0.43	6.03
At the desk facing the surface of the desk (3)	37.99	25.14	3.49	11.07	0.48	0.42	22.85
On the bed, facing the opposite wall (4)	21.76	14.32	1.94	6.22	0.49	0.42	13.07

Suppl. Table 5. Overview of the irradiance-derived equivalent daylight (D65) illuminances (in lux) for the ambient light at different locations in the room, that is, the situation during the day and between assessments in the evening. Measurements were taken from the observer's point of view. Values were calculated using the luox app[11,12]. For a picture of the setup, please see Suppl. Photo 1.

	Equivalent Daylight Illuminance [EDI; lux]			
	L cones	M cone	S-cone	Melanopsin
At desk with display (1)	8.00	5.80	1.28	2.67
On the sofa facing away from the ambient light (2)	6.17	4.49	0.97	2.07
At the desk facing the surface of the desk (3)	23.32	17.27	4.28	8.35
On the bed, facing the opposite wall (4)	13.36	9.83	2.37	4.69

Suppl. Table 6: Interpretation of Bayes Factors according to Jeffreys (1961).

Bayes Factor	Interpretation	
**BF < 1/100**	**Extreme Evidence**	In favour of H0
**1/30 > BF ≥ 1/100**	**Very strong Evidence**	
**1/10 > BF ≥ 1/30**	**Strong Evidence**	
1/3 > BF ≥ 1/10	Moderate Evidence	
1 > BF ≥ 1/3	Anecdotal Evidence	
BF = 1	*No Evidence*	
1 < BF ≤ 3	Anecdotal Evidence	In favour of H1
3 < BF ≤ 10	Moderate Evidence	
**10 < BF ≤ 30**	**Strong Evidence**	
**30 < BF ≤ 100**	**Very strong Evidence**	
**BF > 100**	**Extreme Evidence**	

Bayes factors in bold are deemed conclusive, all other evidence is inconclusive. Abbreviations: BF = Bayes Factor; H0 = null hypothesis; H1 = alternative hypothesis.

Suppl. Table 7: Shift in DLMO in the three light exposure conditions sampled from the posterior distribution.

Effect	Estimate	SD	95% CI (lower; upper)
Condition mean (intercept)	41.7	53.0	-46.9; 128.0
Background	6.8	6.6	-6.2; 20.6
Blue-dim	-0.8	6.7	-13.9; 12.2
Yellow-bright	-6.0	6.5	-19.6; 6.5

Abbreviations: SD = Standard deviation of the estimate; CI = Credible Interval. For each condition, we report differences from the intercept.

Suppl. Table 8: Melatonin values in the three light exposure conditions sampled from the posterior distribution.

Effect	Estimate	SD	95% CI (lower; upper)
Condition mean (intercept)	18.7	8.0	5.0; 32.0
Background	0.15	0.6	-1.0; 1.3
Blue-dim	0.3	0.6	-0.9; 1.5
Yellow-bright	-0.4	0.6	-1.6; 0.7

Abbreviations: SD = Standard deviation of the estimate; CI = Credible Interval. For each condition, we report differences from the intercept.

Suppl. Table 9: Subjective sleepiness ratings on the Karolinska Sleepiness Scale (KSS) during the light exposure in the three light conditions sampled from the posterior distribution.

**Effect**	**Estimate**	**SD**	**95% CI (lower; upper)**
Condition mean (intercept)	7.77	1.4	5.5; 10.1
Background	-0.01	0.1	-0.01: 0.4
Blue-dim	0.2	0.1	-0.2; 0.2
Yellow-bright	-0.19	0.1	-0.4; 0.02

Abbreviations: SD = Standard deviation of the estimate; CI = Credible Interval. For each condition, we report differences from the intercept.

Suppl. Table 10: Objective sleepiness (i.e., Alpha [8-12 Hz]/ Theta [4-7 Hz] ratio) during the Karolinska Drowsiness Tests (KDT) at the beginning, 30 min into, and at the end of the light exposure in the three light conditions sampled from the posterior distribution.

**Effect**	**Estimate**	**SD**	**95% CI (lower; upper)**
Condition mean (intercept)	1.1	0.42	0.5; 1.8
Background	-0.04	0.03	-0.1; 0.01
Blue-dim	0.004	0.03	-0.05; 0.05
Yellow-bright	0.03	0.03	-0.01; 0.09

Abbreviations: SD = Standard deviation of the estimate; CI = Credible Interval. For each condition, we report differences from the intercept.

Suppl. Table 11: Visual comfort scores during the light exposure in the three light conditions sampled from the posterior distribution.

**Effect**	**Estimate**	**SD**	**95% CI (lower; upper)**
Condition mean (intercept)	2.8	0.8	1.6; 4.0
Background	-0.04	0.06	-0.2; 0.07
Blue-dim	0.07	0.06	-0.04; 0.2
Yellow-bright	-0.03	0.06	-0.1; 0.08

Abbreviations: SD = Standard deviation of the estimate; CI = Credible Interval. For each condition, we report differences from the intercept.

Suppl. Table 12: Median reaction times during the light exposure in the three light conditions sampled from the posterior distribution.

**Effect**	**Estimate**	**SD**	**95% CI (lower; upper)**
Condition mean (intercept)	393.0	25.4	347.4; 438.3
Background	-8.0	2.1	-12.2; -3.8
Blue-dim	6.0	2.1	2.0; 10.1
Yellow-bright	2.0	2.0	-2.0; 6.0

Abbreviations: SD = Standard deviation of the estimate; CI = Credible Interval. For each condition, we report differences from the intercept.

Suppl. Table 13: Fastest 10% reaction times during the light exposure in the three light conditions sampled from the posterior distribution.

**Effect**	**Estimate**	**SD**	**95% CI (lower; upper)**
Condition mean (intercept)	334.1	25.5	298.0; 367.0
Background	-3.6	1.5	-6.6; -0.7
Blue-dim	2.8	1.5	-0.01; 5.8
Yellow-bright	0.7	1.4	-2.0; 3.5

Abbreviations: SD = Standard deviation of the estimate; CI = Credible Interval. For each condition, we report differences from the intercept.

Suppl. Table 14: Slowest 10% reaction times during the light exposure in the three light conditions sampled from the posterior distribution.

**Effect**	**Estimate**	**SD**	**95% CI (lower; upper)**
Condition mean (intercept)	505.0	220.5	394.2; 617.6
Background	-19.5	5.4	-30.0; -9.0
Blue-dim	12.5	5.2	2.5; 22.7
Yellow-bright	7.03	5.1	-3.2; 17.1

Abbreviations: SD = Standard deviation of the estimate; CI = Credible Interval. For each condition, we report differences from the intercept.

Suppl. Table 15: Latency to continuous 10 min of sleep in the three light conditions sampled from the posterior distribution.

**Effect**	**Estimate**	**SD**	**95% CI (lower; upper)**
Condition mean (intercept)	11.36	30.6	-38.1; 60.5
Background	4.13	3.7	-3.05; 11.9
Blue-dim	-2.6	3.7	-10.2; 4.6
Yellow-bright	-1.5	3.6	-8.8; 5.8

Abbreviations: SD = Standard deviation of the estimate; CI = Credible Interval. For each condition, we report differences from the intercept.

Suppl. Table 16: Perceived brightness before the start of the light exposure in the three conditions sampled from the posterior distribution.

Effect	Estimate	SD	95% CI (lower; upper)
Condition mean (intercept)	3.4	0.9	2.5; 4.3
Background	-0.07	0.02	-0.1; -0.03
Blue-dim	-0.02	0.02	-0.1; 0.02
Yellow-bright	0.09	0.02	0.04; 0.1

Abbreviations: SD = Standard deviation of the estimate; CI = Credible Interval. For each condition, we report differences from the intercept.

Suppl. Table 17. Sleep Descriptives.

	Sleep Onset Latency [min]	Latency to 10 min continuous sleep [min]	Sleep Efficiency [%]	Wake after Sleep Onset [min]	Number of Awakenings	N1 Latency [min]	N1 Percent	N2 Latency [min]	N2 Percent	N3 Latency [min]	N3 Percent	REM Latency [min]	REM Percent
**Background**	5.8±2.4	19.6±46.8	92.6±10.5	20.7±36.7	10.9±5.0	5.8±2.4	6.9±2.9	8.7±2.7	44.6±4.8	34.0±44.0	24.8±6.6	111.2±73.6	23.7±4.6
**Blue-dim**	5.7±3.0	7.5±4.2	92.9±6.4	19.5±21.4	9.9±5.5	5.7±3.0	6.3±3.3	8.5±3.8	44.8±6.0	23.3±7.3	24.4±6.0	96.7±49.6	24.5±6.9
**Yellow-bright**	3.9±1.9	10.0±14.2	94.8±3.9	14.7±12.9	11.2±7.0	3.9±1.9	6.8±2.9	7.4±4.1	45.0±5.2	26.1±17.6	24.9±6.2	104.2±78.3	23.3±7.2
**Visit 1**	5.8±2.4	19.6±46.8	92.6±10.5	20.7±36.7	10.9±5.0	5.8±2.4	6.9±2.9	8.7±2.7	44.6±4.8	34.0±44.0	24.8±6.6	111.2±73.6	23.7±4.6
**Visit 2**	5.7±2.7	8.0±5.2	93.2±6.1	18.5±19.9	11.5±6.4	5.7±2.7	7.1±3.1	8.8±4.1	44.0±4.9	23.5±8.0	24.5±5.5	85.5±44.4	24.5±5.6
**Visit 3**	3.9±2.4	9.5±13.9	94.5±4.5	15.7±15.3	9.6±6.0	3.9±2.4	6.0±2.9	7.0±3.7	45.8±6.1	25.8±17.4	24.8±6.6	115.5±78.5	23.4±8.3

Participants had a 6-h sleep opportunity starting 2 hours after habitual bedtime. Values reflect the mean ± the standard deviation. Note that Visit 1 was always the “Background” condition.

Suppl. Table 18: Slow wave activity during the first sleep cycle in the three light conditions sampled from the posterior distribution.

Effect	Estimate	SD	95% CI (lower; upper)
Condition mean (intercept)	106.0	100.4	-49.7; 260.5
Background	-1.2	4.3	-9.6; 7.1
Blue-dim	-2.0	4.3	-10.4; 6.2
Yellow-bright	3.3	4.3	-5.2; 11.8

Abbreviations: SD = Standard deviation of the estimate; CI = Credible Interval. For each condition, we report differences from the intercept.

Suppl. Table 19: Melatonin values before the start of the light exposure in the three light conditions sampled from the posterior distribution.

Effect	Estimate	SD	95% CI (lower; upper)
Condition mean (intercept)	8.2	6.6	-0.9; 17.9
Background	0.3	0.25	-0.2; 0.8
Blue-dim	-0.1	0.25	-0.6; 0.4
Yellow-bright	-0.1	0.25	-0.6; 0.4

Abbreviations: SD = Standard deviation of the estimate; CI = Credible Interval. For each condition, we report differences from the intercept.

Suppl. Table 20: Median reaction times prior to the light exposure in the three light conditions sampled from the posterior distribution.

Effect	Estimate	SD	95% CI (lower; upper)
Condition mean (intercept)	363.2	27.8	316.5; 409.4
Background	-4.1	1.1	-6.3; -1.9
Blue-dim	2.3	1.1	0.1; 4.5
Yellow-bright	1.8	1.1	-0.4; 4.0

Abbreviations: SD = Standard deviation of the estimate; CI = Credible Interval. For each condition, we report differences from the intercept.

Suppl. Table 21: Subjective sleepiness scores before the start of the light exposure in the three light conditions sampled from the posterior distribution.

Effect	Estimate	SD	95% CI (lower; upper)
Condition mean (intercept)	5.7	1.7	3.23; 8.1
Background	0.4	0.1	0.3; 0.6
Blue-dim	-0.3	0.1	-0.4; -0.1
Yellow-bright	-0.2	0.1	-0.3; -0.1

Abbreviations: SD = Standard deviation of the estimate; CI = Credible Interval. For each condition, we report differences from the intercept.

Suppl. Table 22. Systematic overview of exclusion criteria.

Aspect	Modality	Exclusion criterion	Time of assessment
Ability to understand study materials	n/a	Inability to understand study materials	Pre-screening
Previous exposure to study	n/a	Prior participation in study	Pre-screening
Relationship with study team	n/a	Family members, employees or other dependent persons	Pre-screening
Normal body weight	BMI calculated from self-reported height and weight	<18.5 or >24.9	Pre-screening
Pregnancy	Self-report	Pregnancy	Pre-screening
Shift work	Self-report	Shift work <3 months prior to Visit 1	Pre-screening
Travel across time zones	Self-report	Travel across two time zones <1 month prior to Visit 1	Pre-screening
Chronotype	MCTQ	≤2 or ≥7	Pre-screening
Habitual sleep duration	MCTQ	<6 or >10 hours	Pre-screening
Physical health	Physical examination by study physician		Adaptation night (Visit 1)
Medications impacting on visual, neuroendocrine, sleep, and circadian physiology	Physical examination by study physician		Adaptation night (Visit 1)
Sleep efficiency	PSG / Laboratory log	<70% (total sleep time / time in bed)	Adaptation night (Visit 1)
Normal sleep	Self-report	Any indicators of sleep disorder	Adaptation night (Visit 1)
Photosensitive epilepsy	Self-report	Self-reported photosensitive epilepsy	Adaptation night (Visit 1)
Colour vision	Cambridge Colour Test	Normal colour vision	Adaptation night (Visit 1)
Core body temperature	Core body temperature measurements using ingestible pill	Drop less than 0.2 between arrival in the laboratory and habitual bed time	Adaptation night (Visit 1)
Circadian stabilisation	Actigraphy and sleep diary	Deviation of target bed or wake time >30 minutes 2x during 5 days prior to study visit	Prior to experimental visits (Visit 2, 3 and 4)

Suppl. Picture 1. Laboratory setup. Photo of the laboratory setup and illustrations of the four locations, where measurements of ambient illuminance were taken from the observer's point of view. The numbers in the picture correspond to (1) "at desk with display", (2) "on the sofa facing away from the ambient light", (3) "at the desk facing the surface of the desk", and (4) "on the bed, facing the opposite wall". Participants spent most of the time during the day and in the evenings at locations 1 and 2. Written informed consent was obtained from the individual in this photo.

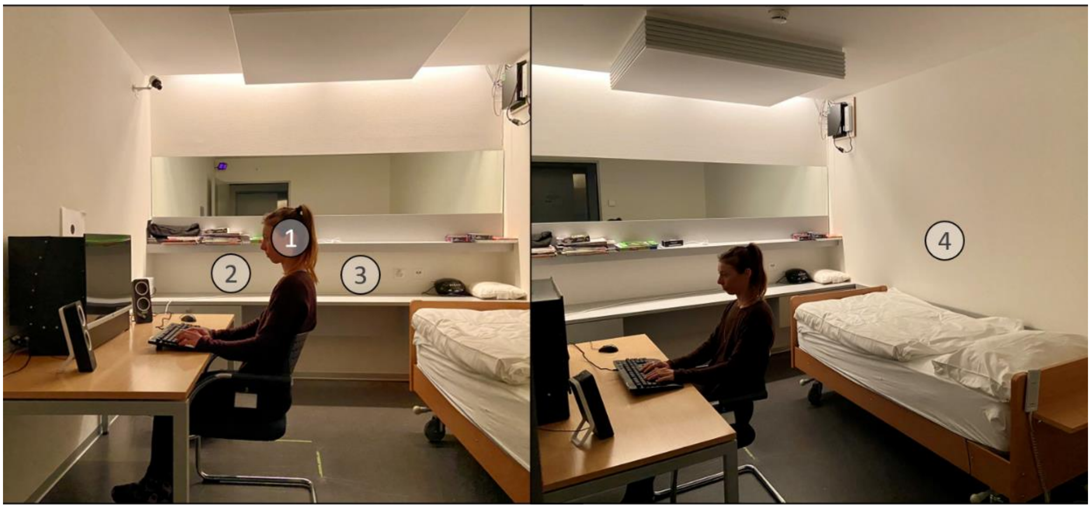

Laboratory Log

Data were collected between 28/02/2022 (first participant's adaptation visit) and 22/12/2022 (last participant's last experimental visit). Below, we specify the date of each visit.

The experimental protocol is detailed in the main manuscript.

**Participant**	**Date Adaptation Night**	**Experimental Visit 1**	**Experimental Visit 2**	**Experimental Visit 3**
1	28-09-2022	05-10-2022	12-10-2022	19-10-2022
2	28-03-2022	04-04-2022	11-04-2022	18-04-2022
3	06-07-2022	13-07-2022	20-07-2022	27-07-2022
4	04-07-2022	11-07-2022	18-07-2022	25-07-2022
5	28-09-2022	05-10-2022	12-10-2022	19-10-2022
6	02-11-2022	09-11-2022	16-11-2022	23-11-2022
7	14-11-2022	21-11-2022	28-11-2022	06-12-2022
8	30-11-2022	07-12-2022	14-12-2022	21-12-2022
9	28-02-2022	14-03-2022	21-03-2022	28-03-2022
10	18-04-2022	25-04-2022	02-05-2022	09-05-2022
11	06-06-2022	13-06-2022	20-06-2022	27-06-2022
12	06-06-2022	13-06-2022	20-06-2022	27-06-2022
13	31-08-2022	07-09-2022	14-09-2022	21-09-2022
14	03-10-2022	10-10-2022	17-10-2022	24-10-2022
15	02-11-2022	09-11-2022	16-11-2022	23-11-2022
16	30-11-2022	07-12-2022	14-12-2022	21-12-2022

Example Protocol

Example protocol for the experimental assessments (assumed habitual bedtime 00:00).

Assumed bed time	00:00													
**Local time**	**Local day**	**Time in protocol**	**Lighting**	**Awake/Sleep**		**Snack**	**Melatonin**	**PVT**	**KSS**	**Visual comfort**	**EEG measurements**	**EEG mounting**	
**Beginning of protocol**													
17:00	1	-0.50	Dim light	Awake	Arrival, get changed, drug test								
17:30	1	0.00	Dim light	Awake		0.25*REE						x	
18:00	1	0.50	Dim light	Awake								x	REE =Resting energy expenditure
18:30	1	1.00	Dim light	Awake								x	
19:00	1	1.50	Dim light	Awake			x	x	x	x			
19:30	1	2.00	Dim light	Awake			x	x	x	x			
20:00	1	2.50	Dim light	Awake			x	x	x	x			
20:30	1	3.00	Dim light	Awake			x	x	x	x			
21:00	1	3.50	Dim light	Awake			x	x	x	x			
21:30	1	4.00	Dim light	Awake			x	x	x	x			
22:00	1	4.50	Dim light	Awake			x	x	x	x			
22:30	1	5.00	Dim light	Awake			x	x	x	x			
23:00	1	5.50	Dim light	Awake			x	x	x	x			
23:30	1	6.00	Dim light	Awake			x	x	x	x			
00:00	2	6.50	Dim light	Awake			x	x	x	x			
00:30	2	7.00	Experimental light	Awake			x	x	x	x	KDT		
01:00	2	7.50	Experimental light	Awake			x	x	x	x	KDT		
01:30	2	8.00	Dim light	Awake			x	x	x	x	KDT		
02:00	2	8.50	No light	Sleep							PSG		
02:30	2	9.00	No light	Sleep							PSG		
03:00	2	9.50	No light	Sleep							PSG		
03:30	2	10.00	No light	Sleep							PSG		
04:00	2	10.50	No light	Sleep							PSG		
04:30	2	11.00	No light	Sleep							PSG		
05:00	2	11.50	No light	Sleep							PSG		
05:30	2	12.00	No light	Sleep							PSG		
06:00	2	12.50	No light	Sleep							PSG		
06:30	2	13.00	No light	Sleep							PSG		
07:00	2	13.50	No light	Sleep							PSG		
07:30	2	14.00	No light	Sleep							PSG		
08:00	2	14.50	Dim light	Awake		0.17*REE	x		x				
08:30	2	15.00	Dim light	Awake			x		x				
09:00	2	15.50	Dim light	Awake			x		x				
09:30	2	16.00	Dim light	Awake			x		x				
10:00	2	16.50	Dim light	Awake									
10:30	2	17.00	Dim light	Awake		0.17*REE							
11:00	2	17.50	Dim light	Awake									
11:30	2	18.00	Dim light	Awake									
12:00	2	18.50	Dim light	Awake									
12:30	2	19.00	Dim light	Awake									
13:00	2	19.50	Dim light	Awake		0.17*REE							
13:30	2	20.00	Dim light	Awake									
14:00	2	20.50	Dim light	Awake									
14:30	2	21.00	Dim light	Awake									
15:00	2	21.50	Dim light	Awake									
15:30	2	22.00	Dim light	Awake		0.17*REE							
16:00	2	22.50	Dim light	Awake									
16:30	2	23.00	Dim light	Awake									
17:00	2	23.50	Dim light	Awake									
17:30	2	24.00	Dim light	Awake									
18:00	2	24.50	Dim light	Awake		0.17*REE							
18:30	2	25.00	Dim light	Awake									
19:00	2	25.50	Dim light	Awake			x	x	x				
19:30	2	26.00	Dim light	Awake			x	x	x				
20:00	2	26.50	Dim light	Awake			x	x	x				
20:30	2	27.00	Dim light	Awake		0.17*REE	x	x	x				
21:00	2	27.50	Dim light	Awake			x	x	x				
21:30	2	28.00	Dim light	Awake			x	x	x				
22:00	2	28.50	Dim light	Awake			x	x	x				
22:30	2	29.00	Dim light	Awake			x	x	x				
23:00	2	29.50	Dim light	Awake			x	x	x				
23:30	2	30.00	Dim light	Awake			x	x	x				
00:00	3	30.50	Dim light	Awake			x	x	x				
00:30	3	31.00	Dim light	Awake			x	x	x				
01:00	3	31.50	Dim light	Awake			x	x	x				
01:30	3	32.00	Dim light	Awake			x	x	x				
02:00	3	32.50	Dim light	Awake									
End of protocol													

Supplementary References

1 van Doorn, J. *et al.* The JASP guidelines for conducting and reporting a Bayesian analysis. *Psychonomic Bulletin & Review* **28**, 813-826 (2021). https://doi.org:10.3758/s13423-020-01798-5

2 Mouland, J. W., Martial, F., Watson, A., Lucas, R. J. & Brown, T. M. Cones Support Alignment to an Inconsistent World by Suppressing Mouse Circadian Responses to the Blue Colors Associated with Twilight. *Curr Biol* **29**, 4260-4267 e4264 (2019). https://doi.org:10.1016/j.cub.2019.10.028

3 Dacey, D. M. *et al.* Melanopsin-expressing ganglion cells in primate retina signal colour and irradiance and project to the LGN. *Nature* **433**, 749-754 (2005). https://doi.org:10.1038/nature03387

4 Patterson, S. S., Kuchenbecker, J. A., Anderson, J. R., Neitz, M. & Neitz, J. A Color Vision Circuit for Non-Image-Forming Vision in the Primate Retina. *Current Biology* (2020). https://doi.org:10.1016/j.cub.2020.01.040

5 Krauskopf, J., Williams, D. R. & Heeley, D. W. Cardinal directions of color space. *Vision Res* **22**, 1123-1131 (1982). https://doi.org:10.1016/0042-6989(82)90077-3

6 Webster, M. A. & Mollon, J. D. The influence of contrast adaptation on color appearance. *Vision Res* **34**, 1993-2020 (1994). https://doi.org:10.1016/0042-6989(94)90028-0

7 Spitschan, M., Jain, S., Brainard, D. H. & Aguirre, G. K. Opponent melanopsin and S-cone signals in the human pupillary light response. *Proc Natl Acad Sci U S A* **111**, 15568-15572 (2014). https://doi.org:10.1073/pnas.1400942111

8 Cao, D., Nicandro, N. & Barrionuevo, P. A. A five-primary photostimulator suitable for studying intrinsically photosensitive retinal ganglion cell functions in humans. *J Vis* **15**, 15 11 27 (2015). https://doi.org:10.1167/15.1.27

9 Woelders, T. *et al.* Melanopsin- and L-cone-induced pupil constriction is inhibited by S- and M-cones in humans. *Proc Natl Acad Sci U S A* **115**, 792-797 (2018). https://doi.org:10.1073/pnas.1716281115

10 Figueiro, M. G., Bullough, J. D., Parsons, R. H. & Rea, M. S. Preliminary evidence for spectral opponency in the suppression of melatonin by light in humans. *Neuroreport* **15**, 313-316 (2004). https://doi.org:10.1097/00001756-200402090-00020

11 Spitschan, M., Nam, S. & Veitch, J. A. luox: Platform for calculating quantities related to light and lighting (2022).https://luox.app.

12 Spitschan, M. *et al.* luox: validated reference open-access and open-source web platform for calculating and sharing physiologically relevant quantities for light and lighting. *Wellcome Open Res* **6**, 69 (2021). https://doi.org:10.12688/wellcomeopenres.16595.3
