# Supplementary material for: Effects of calibrated blue–yellow changes in light on the human circadian clock
Source: Nat Hum Behav. 2023 Dec 22;8(3):590–605. doi: 10.1038/s41562-023-01791-7 (PMC10963261; doi:10.1038/s41562-023-01791-7)
Supplement: Supplementary file 2 — Reporting Summary [file 41562_2023_1791_MOESM2_ESM.pdf]

nature portfolio

Corresponding author(s): Dr. Christine Blume
Prof. Manuel Spitschan

Last updated by author(s): Sep 29, 2023

Reporting Summary

Nature Portfolio wishes to improve the reproducibility of the work that we publish. This form provides structure for consistency and transparency in reporting. For further information on Nature Portfolio policies, see our Editorial Policies and the Editorial Policy Checklist.

Statistics

For all statistical analyses, confirm that the following items are present in the figure legend, table legend, main text, or Methods section.

n/a	Confirmed	
☐	☒	The exact sample size (*n*) for each experimental group/condition, given as a discrete number and unit of measurement
☐	☒	A statement on whether measurements were taken from distinct samples or whether the same sample was measured repeatedly
☐	☒	The statistical test(s) used AND whether they are one- or two-sided<br>*Only common tests should be described solely by name; describe more complex techniques in the Methods section.*
☐	☒	A description of all covariates tested
☐	☒	A description of any assumptions or corrections, such as tests of normality and adjustment for multiple comparisons
☐	☒	A full description of the statistical parameters including central tendency (e.g. means) or other basic estimates (e.g. regression coefficient) AND variation (e.g. standard deviation) or associated estimates of uncertainty (e.g. confidence intervals)
☒	☐	For null hypothesis testing, the test statistic (e.g. *F*, *t*, *r*) with confidence intervals, effect sizes, degrees of freedom and *P* value noted<br>*Give P values as exact values whenever suitable.*
☐	☒	For Bayesian analysis, information on the choice of priors and Markov chain Monte Carlo settings
☒	☐	For hierarchical and complex designs, identification of the appropriate level for tests and full reporting of outcomes
☒	☐	Estimates of effect sizes (e.g. Cohen's *d*, Pearson's *r*), indicating how they were calculated

Our web collection on statistics for biologists contains articles on many of the points above.

Software and code

Policy information about availability of computer code

Data collection	- Screening for normal colour vision: Cambridge Colour Test46 (trivector version) implemented using an iMac-based Metropsis system (Cambridge Research Systems, Rochester, UK)<br>- EEG data collection: BrainVision Recorder Software (BrainProducts GmbH, Gilching, Germany)<br>- Psychomotor Vigilance Task (PVT) and administration of questionnaires (i.e., Karolinska Sleepiness Scale [KSS], visual comfort): Python version 3.6 (Python Software Foundation) using PsychoPy version 3.1.5.<br>- Control of the LEDs in the custom-made display: Q Light Controller + software for DMX control (https://www.qlcplus.org/)
Data analysis	- EEG analyses: EEG raw data were analysed using the Fieldtrip toolbox (Oostenveld et al., 2010; distribution from https://gitlab.com/obob/obob_ownft) running on MATLAB 2022a (The Mathworks, Natick, MA, USA).<br>- Sleep staging: Philips Respironics Sleepware G3 software version 4.0.1.0.<br>- Dim Light Melatonin Onset: Hockey-stick algorithm version 2.5 (cf. Danilenko et al., 2014)<br>- Statistical analyses: R version 4.2.3 using the BayesFactor package (all analysis codes are available here https://github.com/ChristineBlume/Effects-of-calibrated-blue-yellow-changes-in-light-on-the-human-circadian-clock/)

For manuscripts utilizing custom algorithms or software that are central to the research but not yet described in published literature, software must be made available to editors and reviewers. We strongly encourage code deposition in a community repository (e.g. GitHub). See the Nature Portfolio guidelines for submitting code & software for further information.

Data

Policy information about availability of data

All manuscripts must include a data availability statement. This statement should provide the following information, where applicable:

- Accession codes, unique identifiers, or web links for publicly available datasets
- A description of any restrictions on data availability
- For clinical datasets or third party data, please ensure that the statement adheres to our policy

All data generated in this study (including laboratory logs) are available in anonymised and deidentified form on FigShare (Data: https://doi.org/10.6084/m9.figshare.23578698; Laboratory Log: https://doi.org/10.6084/m9.figshare.23578695

Research involving human participants, their data, or biological material

Policy information about studies with human participants or human data. See also policy information about sex, gender (identity/presentation), and sexual orientation and race, ethnicity and racism.

Reporting on sex and gender	Sex was determined based on self-reporting. Note that in German, there is only one word for sex/gender ("Geschlecht"), which is commonly interpreted as "sex". We collected data from an equal number of men and women and sex was a control variable (i.e., random effect, termed "gender" in the codes as it was self-reported) in our analyses. In the data that we publish with this manuscript, sex is included at the participant level (m = male vs. f = female).
Reporting on race, ethnicity, or other socially relevant groupings	As we did not expect social variables, race or ethnicity to affect our results, we did not have exclusion criteria relating to such variables. Thus, we do not expect that there was a relevant social grouping effect and we have no record of such variables. The only control variable that was included in the analyses was sex.
Population characteristics	Participants were young and healthy both mentally and physically (cf. exclusion criteria). The age range was limited to 18-35, which resulted in a mean age of 25.5±2.7 years. An equal number of men and women was included in the study.
Recruitment	Participants were recruited through an ad on a website run by the University of Basel, which includes a job board (www.markt.unibas.ch).
Ethics oversight	Approval for this study was granted from the Ethikkommission Nordwest- und Zentralschweiz (EKNZ) with approval number 2020-02037.

Note that full information on the approval of the study protocol must also be provided in the manuscript.

Field-specific reporting

Please select the one below that is the best fit for your research. If you are not sure, read the appropriate sections before making your selection.

☒ Life sciences ☐ Behavioural & social sciences ☐ Ecological, evolutionary & environmental sciences

For a reference copy of the document with all sections, see nature.com/documents/nr-reporting-summary-flat.pdf

Life sciences study design

All studies must disclose on these points even when the disclosure is negative.

Sample size	Assuming a large effect size (ES; Cohen's d = 0.8) and that H1 better predicts the data than H0, Bayes Factor Design simulations revealed that with 16 participants 62% of the simulations showed evidence for H1 with 38% being inconclusive (medium ES: 22.2% vs. 77.7%; small ES: 0.5% vs. 96.4% and 3.1% showing evidence for H0). However, we argue that, if the S–(L+M) opponent system indeed exerts a very strong effect on the circadian system, then this should be visible on a single-subject level already. In fact, previous research suggests that the assumptions about effect sizes (i.e., d = 0.8) for the circadian phase shifts used in the simulations outlined above may be rather conservative. Our financially driven resource limit was thus n=16 participants.
Data exclusions	No data were excluded from the analyses.
Replication	There was no internal replication. However, we have provided as much detail as possible and necessary to replicate the experiment.
Randomization	All participants underwent all study conditions. The order of the conditions was partly randomised with two possible orders. Participants were allocated to the order by participant number (even vs. uneven number).
Blinding	Blinding was not possible, because the experimenters had to select the correct light exposure and the light exposure conditions were visually distinguishable.

Reporting for specific materials, systems and methods

We require information from authors about some types of materials, experimental systems and methods used in many studies. Here, indicate whether each material, system or method listed is relevant to your study. If you are not sure if a list item applies to your research, read the appropriate section before selecting a response.

Materials & experimental systems

n/a	Involved in the study
☒	☐ Antibodies
☒	☐ Eukaryotic cell lines
☒	☐ Palaeontology and archaeology
☒	☐ Animals and other organisms
☐	☒ Clinical data
☒	☐ Dual use research of concern
☒	☐ Plants

Methods

n/a	Involved in the study
☒	☐ ChIP-seq
☒	☐ Flow cytometry
☒	☐ MRI-based neuroimaging

Clinical data

Policy information about clinical studies

All manuscripts should comply with the ICMJE guidelines for publication of clinical research and a completed CONSORT checklist must be included with all submissions.

Clinical trial registration

DRKS00023603

Study protocol

The approved Stage 1 protocol can be found here (https://springernature.figshare.com/articles/journal_contribution/Effects_of_calibrated_blue-yellow_S_L_M_S_L_M_changes_in_light_on_the_human_circadian_clock_Registered_Report_Stage_1_Protocol_/13050215). For any deviations from this original protocol, please see the "Deviations from Protocol" section in the manuscript. A table of the laboratory protocol can be found here (https://figshare.com/articles/dataset/Protocol/23578704).

Data collection

Data acquisition took place continuously between March and December 2022 at the facilities of the Centre for Chronobiology of the University of Basel with a break of 4 weeks in August 2022 (for more details on the distribution of participants across the acquisition period incl. subjectively reported light history on the day of the experimental visit, please see the supplemental material S3 and the laboratory log). Participants always entered the lab on the same day of the week.

Outcomes

The primary outcome measure was differences in dim light melatonin onset (DLMO) between evenings 1 and 2 of each experimental visit. The DLMO was assessed using the Hockeystick method (version 2.5; Danilenko et al., 2014).

The following secondary outcome measures were assessed:
- Melatonin concentrations were assessed with saliva samples every 30 min starting 5 hours prior to habitual bedtime (HBT) until 1h 30 min after HBT. The samples were assayed by Novolytix GmbH (Pfeffingen, Switzerland) with radioimmunoassays (RIA).
- Subjective sleepiness was assessed with the Karolinska Sleepiness Scale every 30 min starting 5 hours prior to HBT until 1h 30 min after HBT (Akerstedt & Gilberg, 1990; Nordin et al., 2013).
- Objective sleepiness was assessed using EEG-derived alpha (8-12 Hz)/theta (4-7 Hz) ratios assessed during resting-state EEG measurements (3 min, eyes open) before, 30 min into, and after light exposure at electrodes P3, Pz, P4, O1, Oz, O2.
- Visual comfort was assessed using 5-point Likert scales. Participants rated visual comfort every 30 min starting 5 hours prior to HBT until 1h 30 min after HBT. Visual comfort was calculated as the average rating from the responses to the questions about how pleasant the room lighting was generally, how pleasant the brightness was, how glaring the artificial light was, and how pleasant participants rated the colour temperature.
- PVT measures comprised median reaction time, slowest 10% reaction times, and fastest 10% reaction times during a 10-min PVT that was administered every 30 min starting 5 hours prior to HBT until 1h 30 min after HBT. All RTs < 100 ms were dismissed as invalid trials.
- EEG-derived sleep onset latency to 10 min of continuous sleep were based on sleep staging with the Philips Respironics Sleepware G3 software v. 4.0.1.0.
- EEG-derived slow wave activity (SWA) during the first sleep cycle was assessed as the averaged power between 0.5 and 4.5 Hz at electrodes F3, F4, Fz. We further separated the first sleep cycles into percentiles using the "SleepCycle" package for R (Blume & Cajochen, 2021; https://doi.org/10.1016/j.mex.2021.101318).
- Brightness was assessed using a 5-point Likert scale. Participants rated perceived brightness every 30 min starting 5 hours prior to HBT until 1h 30 min after HBT.
